# Phototransformation of Three Psychoactive Drugs in Presence of Sedimental Water Extractable Organic Matter

**DOI:** 10.3390/molecules26092466

**Published:** 2021-04-23

**Authors:** Cristina Jiménez-Holgado, Vasilios Sakkas, Claire Richard

**Affiliations:** 1 Laboratory of Analytical Chemistry, Department of Chemistry, School of Sciences, University of Ioannina, 45110 Ioannina, Greece; crijimhol@gmail.com (C.J.-H.); vsakkas@uoi.gr (V.S.); 2 Institute of Chemistry of Clermont-Ferrand, Université Clermont Auvergne, CNRS, SIGMA-Clermont, ICCF, F-63000 Clermont-Ferrand, France

**Keywords:** antidepressants, sediment, photodegradation, water extractable organic matter, photoproducts

## Abstract

Psychoactive drugs are classified as contaminants of emerging concern but there is limited information on their fate in surface waters. Here, we studied the photodegradation of three psychoactive drugs (sertraline, clozapine, and citalopram) in the presence of organic matter (WEOM) extracted under mild conditions from sediment of Lake Pamvotis, Greece. Spectral characterization of WEOM confirmed its humic-like nature. Preliminary experiments using chemical probes showed that WEOM was able to produce oxidant triplet excited state (^3^WEOM*), singlet oxygen (^1^O_2_), and hydroxyl radicals under irradiation with simulated solar light. Then, WEOM at 5 mgC L^−1^ was irradiated in the presence of the three drugs. It enhanced their phototransformation by a factor of 2, 4.2, and 16 for sertraline, clozapine, and citalopram, respectively. The drastic inhibiting effect of 2-propanol (5 × 10^−3^ M) on the reactions demonstrated that hydroxyl radical was the key intermediate responsible for drugs photodegradation. A series of photoproducts were identified by ultra-high performance liquid chromatography (UHPLC) coupled to high resolution mass spectrometry (HR-MS). The photodegradation of the three drugs proceeded through several pathways, in particular oxidations of the rings with or without O atom inclusion, N elimination, and substitution of the halogen by OH. The formation of halogenated aromatics was observed for sertraline. To conclude, sedimental natural organic matter can significantly phototransform the studied antidepressant drugs and these reactions need to be more investigated. Finally, ecotoxicity was estimated for the three target analytes and their photoproducts, using the Ecological Structure Activity Relationships (ECOSAR) computer program.

## 1. Introduction

Psychoactive drugs are widely used to treat diseases such as depressive symptoms and social anxiety disorder. This is due to the lack of regulation, together with the ignorance of their fate and the absence of effective methods of elimination, these drugs have been classified as contaminants of emerging concern (CECs). The increasing consumption of these compounds in developed countries enhances in fact the risk of environmental contamination and adverse effects on human health and habitats [1]. They enter the aquatic environment mainly through hospital effluents and wastewater treatment plants and, being poorly degradable by traditional biological processes, they are present in relative high amounts in water bodies and sediments. The psychoactive drugs sertraline (SER), citalopram (CIT), and clozapine (CLO) were detected between 13.2 ng L^−1^ to 15.0 μg L^−1^ in wastewater treatment plants effluents, 8.9 ng L^−1^ to 30.0 μg L^−1^ in fresh waters, and 14.4 to 71.9 ng.g^−1^ in river sediments [2,3,4]. Once released in the aquatic environment, these compounds can undergo chemical reactions and generate new chemicals, that might also be harmful and therefore need to be identified. Although there is no consistent literature data on the ecotoxicity of these compounds, it has been found that acute and chronic exposure of SER and CIT show effects on algae [5], crustaceous [6,7,8], bivalves [9,10] and fish [11]. As well, Villain et al. classified these 3 drugs as class 1 toxicity, where metabolites should be analyzed in priority and the ecotoxicity can be estimated with QSAR models [12]. Calza et al. has found that after 20 min of solar light irradiation, SER and its photoproducts, in the presence of TiO_2_ display higher acute toxicity potential (based on ECOSAR software) [13]).

**Figure 1:**
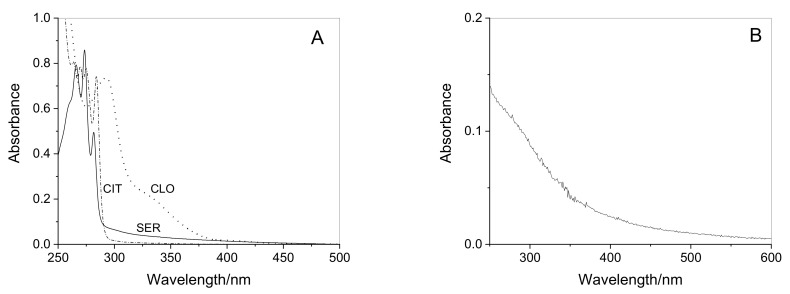
UV-vis absorption of sertraline (SER), clozapine (CLO) and citalopram (CIT) at 5 ppm in water at pH 7 (**A**) and WEOM (18 mgC L^−1^) at pH 9.2 (**B**).

The degradation of these psychoactive drugs can be induced by solar light because they absorb solar radiations between 295 and 450 nm (Figure 1A). According to the literature data, irradiation with simulated solar light in pure water yielded a half-life of 65 d for CIT [14], 8 h for CLO [4] and between 6 min at pH 12 and > 1 h at pH 5 for SER [15]. The phototransformation of SER and CIT were also reported to be sensitized by the natural organic matter, NOM, present in surface water [13,14,15,16]. Indeed, NOM that contains humified light-absorbing compounds can photoinduce the degradation of chemicals through the generation of reactive species such as hydrogen peroxide (H_2_O_2_) and superoxide radical anion (O_2^−^_) [17], ^1^O_2_ [18,19], oxidant triplet excited state (^3^NOM^*^) [20], and hydroxyl radical (HO^.^) [21,22,23].

Part of NOM present in the water column of lakes and rivers comes from sediments resolubilization. Sediments are formed by accumulation of deposited particulate organic matter, that undergoes biochemical transformations [24]. Sediment organic matter generally contains a high proportion of humified compounds and can potentially sensitize the photodegradation of aquatic contaminants. The water-soluble sediment organic matter can be recovered by sampling the sedimental pore water [25], or by extraction from sediment using neutral or slightly alkaline water as described for soils [26].

In this work, we aimed to better understand the fate of SER, CLO and CIT psychoactive drugs when irradiated in the presence of natural organic matter extracted from the Lake Pamvotis (Ioannina, NW Greece) sediment. The water soluble sedimental organic matter (WEOM), beforehand extracted with water under mild conditions, was first characterized by spectral techniques to confirm its humic-like nature. Then, using the scavenging technique, we investigated its capacity to generate reactive species (^3^WEOM^*^, ^1^O_2_ and HO^.^) to finally demonstrate the important role of HO^.^ in the photodegradation of the three drugs. The drugs photoproducts were identified by means of UHPLC-HR-MS and photodegradation pathways were proposed. A toxicity assessment was followed based on the ECOSAR computer model for the parent molecules and their by-products.

## 2. Results and Discussion

### 2.1. Main Characteristics of the Sediments

Sediment was analyzed prior to drugs adsorption measurements. The sediment particle size was found to be equal to 100 ± 10 μm and the percentage of organic matter to 1.91 ± 0.02% of the dry matter. In the literature, the averaged grain size and organic matter content in lake, river and sea sediments vary between silt (2–500 μm) and clay (<2 μm) composition, and contain between 0.37–8.0% of organic matter [27]. Lake Pamvotis is a shallow, closed and eutrophic Mediterranean Lake with ecological significance [28,29]. This lake has been shown to exist since the Pleistocene period [30]. In a previous work, the organic matter content in Lake Pamvotis sediments was found to vary between 4.8% and 15.3%, where the lowest content was found in the coarser sandy sediments and the highest in the silt-clay portion [31]. Our sediments showed a grain size larger than that of previous studies, and the organic matter content obtained was therefore lower, in agreement with previous publications [32,33].

### 2.2. Extraction and Characterization of WEOM from Sediments

By stirring sediment (120 g L^−1^) in pH 9.2 water during 5 d, we could recover WEOM. The DOC content of the WEOM solution was of 18 mgC L^−1^. WEOM showed the typical UV-visible absorption spectrum of humic-like substances with an exponentially decreasing absorption extending up to 600 nm with a shoulder around 280 nm (Figure 1B). SUVA_254_ was equal to 0.44 L.mg^−1^ m^−1^, S_275-295_ to 0.0105 nm^−1^ and E_2_/E_3_ to 3.41. From the SUVA_254_ and E_2_/E_3_ values, we could estimate that the percentage of aromaticity was equal to 6.5% and the average Mw to 1.7 kDa. These data that characterize an organic matter moderately aromatic with an overall small molecular weight are in line with those of other sediment organic matters [25,34]. Among main ions detected in Lake Pamvotis, nitrate was the highest concentrated. UHPLC-MS analysis revealed that nitrate (*m*/*z* = 61.9873 at ±5 ppm) was present in WEOM at a concentration <5 × 10^−5^ M, therefore too low for a significant photochemical effect.

**Figure 2:**
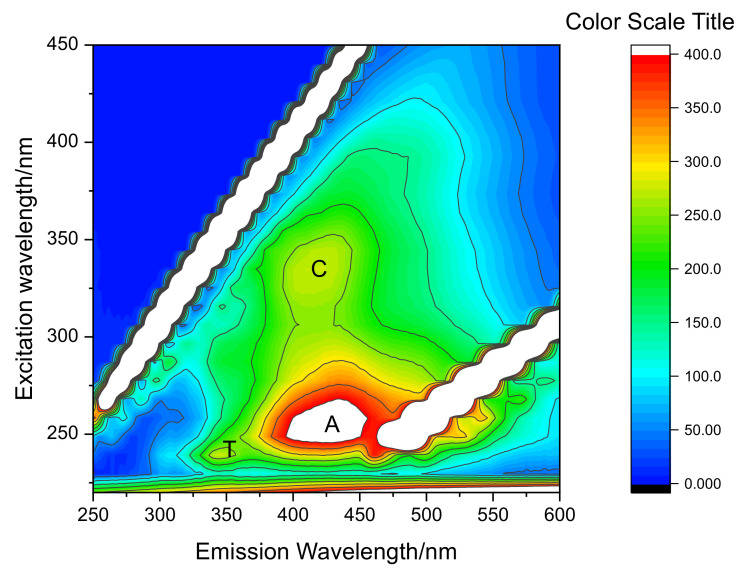
Three dimensional fluorescence spectrum of WEOM (18 mgC L^−1^) at pH 9.2.

The three-dimensional fluorescence spectrum of WEOM contained peaks A and C, assigned as UVC and UVA humic-like fluorophores, respectively, and peaks T assigned as protein tryptophan-like fluorophores (Figure 2) [35]. All these spectral characteristics confirmed the humic-like nature of WEOM. The values of fluorescence indices FI (1.47) and BIX (0.79) that represent the relative contribution of terrestrial and microbial DOM sources and the relative contribution of autochthonous natural organic matter, respectively, indicated that sediments from Lake Pamvotis had a terrestrial contribution, as supported by previous publications [36].

### 2.3. Photoproduction of Reactive Species upon WEOM Irradiation

Three chemical probes (2,4,6-trimethylphenol, furfuryl alcohol and terephthalic acid) were used to evidence the production of photooxidants ^3^WEOM^*^, ^1^O_2_ and HO^.^, respectively [37]. While it is photostable when irradiated alone, 2,4,6-trimethylphenol (5 × 10^−6^ M) disappeared in the presence of WEOM (5 mgC L^−1^) at circumneutral pH in accordance with the formation of ^3^WEOM^*^ (Supplementary Figure S3). The apparent first order rate constant of reaction k was equal to 0.020 ± 0.002 min^−1^ (Supplementary Figure S1). The quantum yield coefficient of the phenol photodegradation f_TMP_ was equal to k/R_a_^WEOM^, where R_a_^WEOM^ was the rate of light absorption by WEOM in the reactor. One found f_TMP_ = 46 ± 5 M^−1^, a value falling in the range of those reported for other NOMs [38].

WEOM was also expected to generate ^1^O_2_ because this species is produced from the deactivation of ^3^WEOM^*^ by oxygen. The loss of furfuryl alcohol (5 × 10^−5^ and 10^−4^ M) upon irradiation in the presence of WEOM (5 mgC L^−1^) confirmed this hypothesis (Supplementary Figure S2). From the k value (0.0011 ± 0.0002 min^−1^), one could estimate the quantum yield of ^1^O_2_ formation, Φ_SO_, using the simplified relationship: Φ_SO_ = α_FFA_ × k × (Furfuryl alcohol)/R_a_^WEOM^, where α_FFA_ is the fraction of ^1^O_2_ scavenged by furfuryl alcohol. Considering that ^1^O_2_ reacts with furfuryl alcohol with a bimolecular rate constant of 1.2 × 10^8^ M^−1^ s^−1^ [39] and is deactivated in water with a monomolecular rate constant of 2.5 × 10^5^ s^−1^ [40], it comes that α_FFA_ was equal to 2.3% and 4.6% by furfuryl alcohol at 5 × 10^−5^ and 10^−4^ M, respectively. This gives Φ_SO_ = 0.055 ± 0.010, a value in the upper limit of those reported for aquatic NOMs [19].

We also irradiated terephthalate (10^−5^ M) in the presence of WEOM (5 mgC L^−1^) at pH = 7. As expected, we observed the formation of the fluorescent hydroxylated photoproduct resulting from the reaction between terephthalic acid and HO^.^ radicals (4.4 × 10^9^ M^−1^ s^−1^ [41]) (Supplementary Figure S3). Using a calibration curve (Supplementary Figure S3), one could determine that the hydroxylated photoproduct was formed at a rate of (5.9 ± 0.6) × 10^−11^ M s^−1^ during the first 40 min of the reaction.

**Scheme 1:**
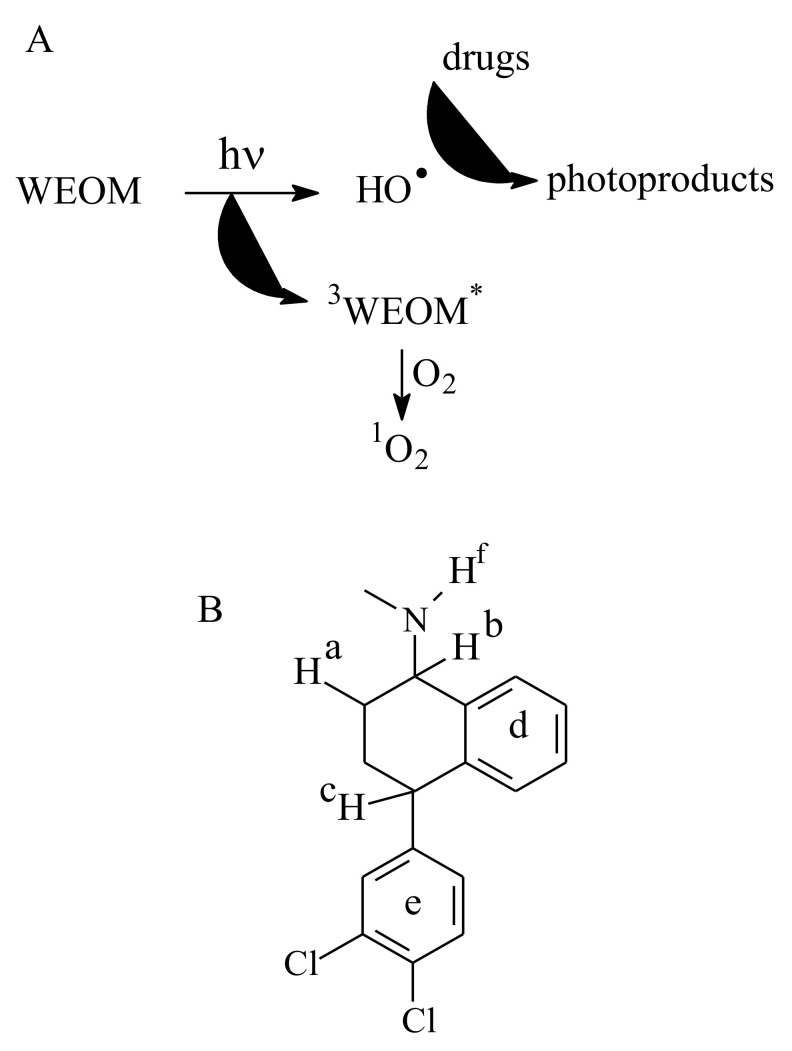
Reactive species production from WEOM (**A**) and chemical structure of SER (**B**).

Therefore, as for other NOMs of water and soils, WEOM of Lake Pamvotis sediment was able to generate photooxidants under simulated solar light irradiation (Scheme 1A).

### 2.4. Irradiation of Drugs in the Presence of WEOM

**Figure 3:**
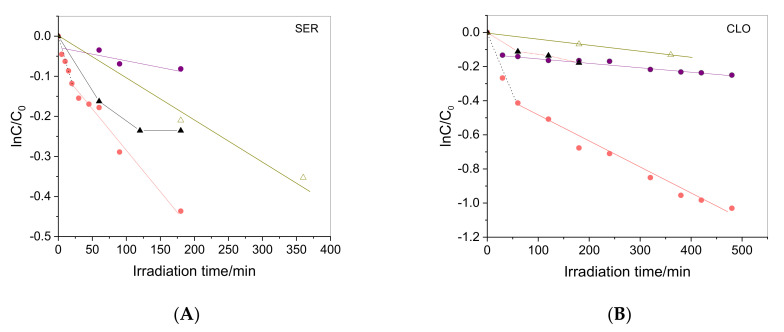

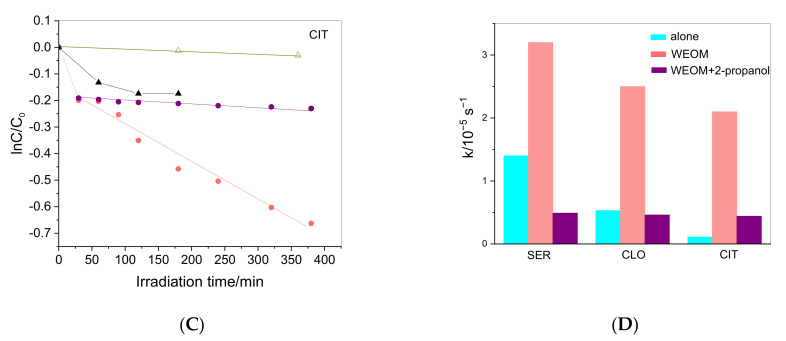
Photodegradation of SER (**A**), CLO (**B**) and CIT (**C**) (5 × 10^−6^ M) in purified water buffered at pH 7 (△), in the presence of WEOM (5 mgC L^−1^) (●) and in the presence of WEOM (5 mgC L^−1^) and 2-propanol (5 × 10^−3^ M) (●). Dark control experiments (▲). Apparent first order of disappearance of drugs in different conditions (**D**).

The drugs (5 × 10^−6^ M) were first irradiated in pH 7 buffered purified water to measure their rate of direct photolysis. Plots of lnC/C_0_ vs. irradiation time are presented in Figure 3A–C. The rate constants deduced from these linear plots (k_direct photolysis_) were of (1.6 ± 0.2) × 10^−5^, (0.60 ± 0.03) × 10^−5^ and (1.3 ± 0.1) × 10^−6^ s^−1^ for SER, CLO and CIT, respectively (Figure 3D and Supplementary Table S1). This order of photoreactivity is consistent with the literature data, where SER was reported to be faster photolyzed than the two other drugs [4,14,15].

Then, drugs were irradiated in the presence of WEOM (5 mgC L^−1^) (Figure 3A–C). For all of them, the plot of lnC/C_0_ vs. irradiation time presented two parts. The slope of the first linear part (between 0 and 20 to 50 min) was higher than that of the second linear part. Such a two-part photodegradation curve was neither observed with 2,4,6-trimethylphenol (Supplementary Figure S1) nor with furfuryl alcohol (Supplementary Figure S2). Dark control experiments revealed that drugs disappeared even in the absence of light—likely by adsorption on the small particles <0.45 μm present in WEOM solutions filtrated with 0.45 μm filters. Accordingly, the three drugs showed significant adsorption on the sediment (Supplementary Figure S4). Therefore, the first part of the plots was not taken into account for the kinetic study and the rate constants k were extracted from the second part of the plots exclusively. The k values in the presence of WEOM varied between 2.1 and 3.5 × 10^−5^ s^−1^ (Figure 3D). In the presence of WEOM, drugs were supposed to disappear by direct photolysis and by WEOM-mediated photodegradation and k was thus equal to:

k = k_direct photolysis*_ + k_WEOM_

where k_direct photolysis*_ was k_direct photolysis_ after correction for the screen effect of WEOM (SI, Text 1), and k_WEOM_ is the rate constant due to the sole contribution of WEOM. Values of k_WEOM_ were obtained by subtracting k_direct photolysis*_ from k. Values of k_WEOM_ varied within a very narrow range (1.8–2.0 × 10^−5^ s^−1^) (Supplementary Table S1) in line with an important contribution of HO^.^ radical in the reactions because these radicals show poor specificity and were expected to oxidize the three drugs at the same rate.

To confirm the involvement of HO^.^ radical, drugs were irradiated in the presence of WEOM (5 mgC L^−1^) and 2-propanol (5 × 10^−3^ M) used as an HO^.^ radical quencher (k_HO_ = 1.9 × 10^9^ M^−1^ s^−1^ [42]). All the rates of disappearance were drastically reduced (Figure 3A–C) and for each drug the rate constant obtained in the presence of WEOM and 2-propanol (k_2-propanol_) approached k_direct photolysis*_ indicating the very high contribution of HO^.^ radical in the WEOM photosensitized reaction (Figure 3D, Supplementary Table S1). In the case of SER, k_2-propanol_ was even lower than k_direct photolysis*_.

### 2.5. Drugs Photoproducts

**Figure 4:**
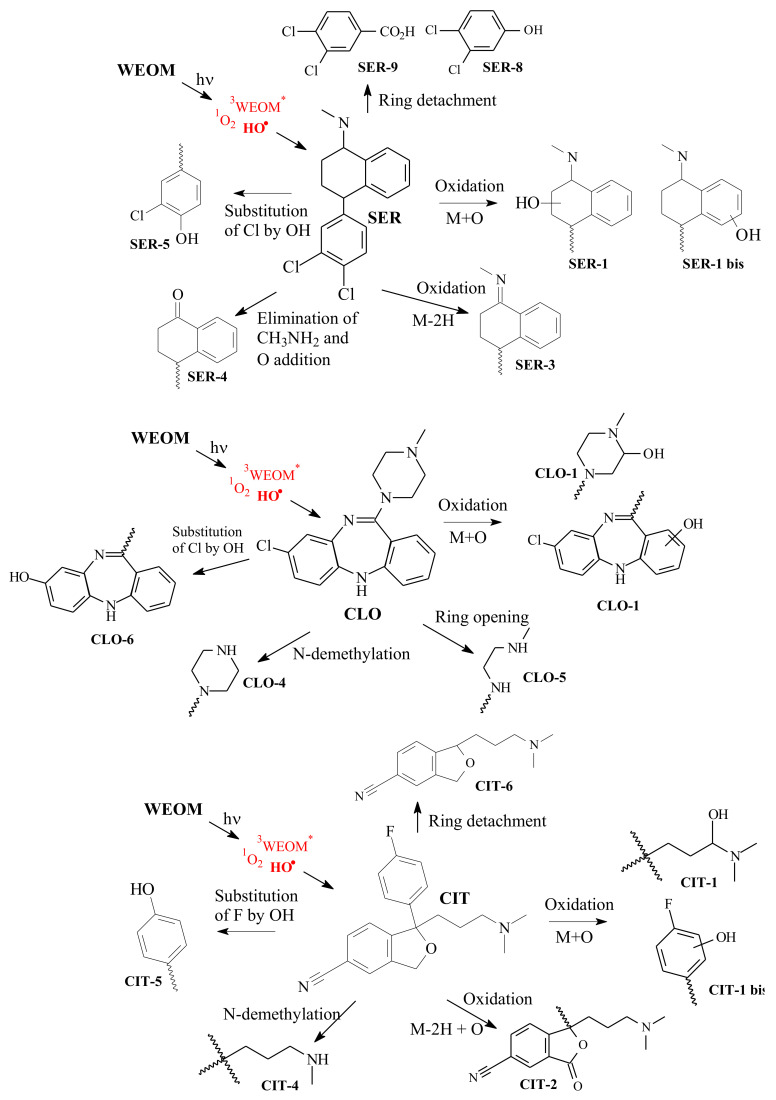
Photodegradation pathways.

Photoproduct analysis was performed on neutral solutions of drugs (5 × 10^−6^ M) and WEOM (5 mgC L^−1^) irradiated for 8 h until drugs conversion between 33% and 69%. UHPLC-MS data are compiled in Table 1, Table 2 and Table 3 while main pathways deduced from MS data are given in Figure 4.

Most of the peaks were detected in positive mode. Molecular ion clusters at *m*/*z* = 322.0753, 324.0722, 326.0687 (SER- and SER-1bis), *m*/*z* = 343.1323, 345.1291 (CLO-1) and peak at *m*/*z* = 341.1658 (CIT-1) corresponded all to [M + O + H]^+^. Double O atom addition was also detected for SER and CLO (SER-2, CLO-2) and even triple O atom addition for CLO (CLO-3).

Molecular ion clusters at *m*/*z* = 304.0647, 306.0615, 308.0581 (SER-3) corresponded to [M − 2H + H]^+^ and to the formation of a double bond C = C or C = N. Formation of an imine was already proposed [15,16]. In acidic aqueous solution, the imine is expected to be hydrolyzed into the corresponding carbonyl [43]. SER-4 with *m*/*z* = 291.0330, 293.0300, 0295.0266 corresponding to SER − CH_3_NH_2_ + O, could therefore be formed in two steps, first oxidation with the imine RR’C = N − CH_3_ formation, then imine hydrolysis into the carbonyl RR’C = O. In the case of CIT, the elimination of 2 H atoms and addition of O was observed (CIT-2) in accordance with a carbonyl formation [44].

Substitution of the halogen atoms, F or Cl, by OH was observed for the three drugs: SER-5 with *m*/*z* = 288.1146 and 290.1110, SER-6 with *m*/*z* = 270.1481 [16], CLO-6 with *m*/*z* = 309.1716 and CIT-5 with *m*/*z* = 323.1757. Such nucleophilic substitution was often reported in the photolysis of halogenoaromatics and was already observed for the studied drugs [4,44,45].

Interestingly, analysis in negative mode yielded molecular ion clusters at *m*/*z* = 160.9553, 162.9524, 164.9495 (SER-8) and *m*/*z* = 188.9507, 190.9275, 192.9279 (SER-9) corresponding to 3,4-dichlorophenol and 3,4-dichlorobenzoic acid, respectively. This result demonstrated that ring detachment took place. The formation of these compounds that show potential toxicity as halogenophenols in general was never reported in the literature to the best of our knowledge. For CIT, the peak at *m*/*z* = 231.1492 (CIT-6) may also result from the cleavage of the aromatic ring.

CLO and CIT both underwent N-demethylation as demonstrated by the detection of [M − CH_2_ + H]^+^ ions (CLO-4 and CIT-4), not observed in the case of SER. This reaction was reported by several authors [2,4,14,44] under various oxidation conditions (UV photolysis, photocatalysis, simulated sunlight irradiation in river water).

Last, in the case of CLO, piperazine ring opening probably took place as shown by the detection of molecular ions cluster at *m*/*z* = 301.1216 and 303.1184 (CLO-5) corresponding to [M − C_2_H_2_ + H]^+^. The same compound was observed in the photocatalysis transformation of CLO [4].

Several other photoproducts were found, arising from the combination of the above-described pathways, for instance: SER-7 formed after CH_3_NH_2_ and Cl elimination, CLO-7 after demethylation and dechlorination, and CIT-10 after demethylation and oxidation.

Given the complexity of the drugs structure and the very oxidant properties of HO^.^ radicals, it is highly probable that the attack of drugs by HO^.^ took place on several sites (Scheme 1B). Some of M+O compounds (SER-1 and CIT-1) loose H_2_O easily. It is in line with the presence of CH(OH)-CH_2_ functionalities in the molecule and with the abstraction of H^a^, H^b^ or H^c^, leading to the aliphatic ring oxidation. N-demethylation observed for CLO and CIT were likely due to abstraction of methyl H atoms. HO^.^ radicals could also add to the aromatic rings (d and e in Scheme 1B) to form phenolic structures at the end [2,15,16]. Lastly, the oxidation of the N atom into N-oxide after abstraction of H^f^ seems possible [14,44]. The carbon radical produced after H abstraction can either loose a second H atom and yield a double bond or add O_2_ to generate a peroxyl radical, then an hydroperoxide and further a carbonyl or an alcohol. On the other hand, the addition of HO^.^ on the aromatic ring leads to a ring-HO^.^ adduct and finally to a phenolic compound after abstraction of a ring H-atom elimination. Ring eliminations required the cleavage of a C-C bond and probably involved a complex sequence of processes, the first step of which might have been the abstraction of H^c^ by HO^.^ (Scheme 1B).

### 2.6. Prediction of Ecotoxicity Assessment

The potential acute (LC_50_ and EC_50_) and chronic toxicity (ChV) of the 3 target analytes and their photoproducts were predicted using ECOSAR computer program (version 2.0). Based on the predicted ecotoxicity values, SER, CLO, CIT, and their transformation products (TPs), could be classified according to the system established by the Globally Harmonized System of Classification and Labelling of Chemicals (GHS) [46] (Table 4).

SER has been found as a moderately toxic compound (EC = 20 mg L^−1^) [13]. ECOSAR program predicted that SER and its by-products were very toxic to fish, daphnia and algae, except by-product SER-6, which shows an acute toxicity LC_50_ for fish up to 10.8 mg/L, and is classified as a harmful compound (Table 5). SER-3 in particular was even more toxic than SER. This finding supports the previously reported observation that treatment of drugs by irradiation can generate products of greater toxicity than the parent compound [47].

CLO is considered to be a harmful compound, and most of its TPs formed during the degradation process are much less toxic (Table 6). No standardized acute or chronic assay was found in the literature for CLO.

In the case of CIT, data at Table 7 show that the obtained for the parent molecule for fish was about 4.47 mg L^−1^, while LC_50_ and EC_50_ were much lower for daphnia and green algae (about 0.652 and 0.360 mg L^−1^ respectively). The same behavior has been observed for SER and CLO (Table 5 and Table 6). In the three cases, the values for chronic toxicity estimated by ECOSAR are lower than those for acute toxicity, suggesting that invertebrates are likely the most sensitive species to these TPs.

Based on the predicted ecotoxicity values, SER and its TPs are toxic or very toxic. CIT is considered as a toxic compound and most of the TPs formed are harmful or not harmful. By contrast, CLO is classified as harmful and most of its by-products formed are not harmful.

## 3. Experimental

### 3.1. Sediment Sampling and Analysis

Sediment was collected in Lake Pamvotis in Ionnina (Epirus Region, Greece). Lake Pamvotis is a shallow Mediterranean urban lake and it occupies an area of 22.8 km^2^ with a mean depth of 5 m, and is classified among the few European lakes that are sufficiently old to feature native faunas and floras. It is considered as one of the European biodiversity hot spot and is used for different activities such as recreation, tourism, fishing and irrigation. The regional capital Ioannina to the west and the town of Perama to the north are urban settlements fringing the lake, the remaining of its periphery is composed of farmland. In the form of ditches, two major inflows of surface runoff water occur. One of them drains an agricultural watershed mainly, while the second drains a mixed of urban, rural, agricultural and industrial land use watershed. An outflow to the north-west controls the water level for flood prevention. The climate in the region is continental, with cold, wet winters (<0 °C) and hot, dry summers (>30 °C). Annual precipitation (1.1–1.2 m) approximates the evaporation of the lake surface. Sediments were collected in September 2019 close to the lake bank (depth 3–7 cm) using an Eckman type sampler [48] and were air-dried. The samples were then stainless-steel sieved over 2 mm to remove large detritus and benthic organisms. The average particle size of sediment was measured in wet dispersions using a laser diffraction particle size analyzer Malvern Mastersizer 3000 and the percentage of organic matter was obtained by loss-on-ignition (dry combustion between 400 and 500 °C) during 8 h using a high temperature furnace.

### 3.2. Chemicals and Preparation of Solutions

Clozapine (8-Chloro-11-(4-methyl-1-piperazinyl)-5H-dibenzo[b,e][1,4]-diazepine) was purchased from Sigma-Aldrich (quality level 300). Citalopram (1-[3-(dimethylamino)propyl]-1-(4-fluorophenyl)-3H-2-benzofuran-5-carbonitrile, hydrobromide) and sertraline (1S,4S-4-(3,4-dichlorophenyl)-*N*-methyl-1,2,3,4-tetrahydronaphthalen-1-amine, hydrochloride) were purchased from TCI Tokyo Chemical Industry (Tokyo, Japan). All these drugs had a purity higher than 98%. 2,4,6-Trimethylphenol (certified reference material), furfuryl alcohol (analytical grade), terephthalic acid (98%) and hydroxyterephthalate disodium (97%) were purchased from Sigma-Aldrich and used as received. Acetonitrile and methanol for HPLC were from Carlo-Erba and VWR, respectively. The other reagents were of the highest grade available. Water was purified using a reverse osmosis RIOS 5 and Synergy (Millipore) device (resistivity 18 MΩ.cm, DOC < 0.1 mg L^−1^).

Stock solutions of sertraline (1 × 10^−3^ M) and citalopram (5 × 10^−3^ M) were prepared in purified water while those of clozapine (10^−3^ M) in water-acetonitrile (95-5, *v*/*v*). Solubilization was achieved after 24 h stirring at 400 rpm. Stock solutions of 2,4,6-trimethylphenol (6.2 × 10^−4^ M), furfuryl alcohol (10^−2^ M), 2-propanol (2 M), terephthalic acid (10^−2^ M), and hydroxyterephthalate (10^−3^ M) were prepared in purified water and stored in the refrigerator in amber glass bottles before use. When necessary, solutions were buffered at pH 7 using a mixture of KH_2_PO_4_ and Na_2_HPO_4_.

### 3.3. Water Extractable Organic Matter Extraction

Extraction of WEOM was performed by adding 12 g of sediment in 100 mL of purified water adjusted at pH 9.0–9.5 using NaOH and containing Na_2_HPO_4_ (6.6 × 10^−3^ M). Suspensions were placed in 200 mL amber glass screw-capped bottles, degassed in a stream of N_2_ during 10 min, closed and stirred during 5 d at 500 rpm at room temperature. After that, the suspensions were filtered using a vacuum filtering flask (Millipore system), first with filters of 5 μm (5VPP, Durapore membrane filters, Millipore) and then with filters of 0.45 μm (HA, Nitrocellulose, Millipore). The obtained WEOM aqueous solutions were characterized in terms of dissolved organic carbon (DOC), UV-Vis absorption, and fluorescence. The procedure was repeated 3 times and all the WEOM solutions were pooled.

### 3.4. DOC Analyses

A Shimadzu 5050 TOC analyser was used to measure the DOC content of solutions. Measurements were made in triplicate.

### 3.5. Optical Analyses

UV-visible analyses of WEOM solutions were conducted on a Varian Cary 3 spectrophotometer in a 1-cm cuvette with purified water as a reference. The absorbances were measured from 250 to 600 nm. The spectral slope (S in nm^−1^) was calculated between 275 and 295 nm using Equation (1):

A_λ_ = A_λ0_ × e^−S(λ − λ0)^ (1)

where A_λ_ and A_λ0_ are the absorbances at 295 and 275 nm, respectively [48]. The specific absorption coefficient at 254 nm (SUVA_254_ in L mg^−1^ m^−1^) was calculated by dividing the absorbance at 254 nm by the DOC concentration in mg L^−1^. The ratio E_2_/E_3_ was calculated by dividing the absorbance at 250 nm by the absorbance at 365 nm, where E_2_ and E_3_ are the absorbance at 250 and 365 nm, respectively. The percentage of aromaticity [49] and the average molecular weight (Mw) of WEOM [50] were estimated following Equations (2) and (3), respectively:

percent aromaticity = 6.52 × SUVA_254_ + 3.63 (2)

Mw = 0.315 × e^(4.96/(−1.72 + E2/E3))^ (3)

The three-dimensional fluorescence spectrum was recorded using a Perkin Elmer LS 55 Luminescence Spectrometer fitted with a 1-cm quartz cuvette. The bandwidths were set to 10 nm for excitation and 10 nm for emission. A series of emission scans between 250 and 600 nm were collected over excitation wavelengths between 240 and 450 nm at 10 nm increments. The fluorescence index (FI) was obtained by dividing the emission intensity at 450 nm by the emission intensity at 500 nm for excitation at 370 nm [51] and the biological index (BIX) was calculated as the ratio of emission intensity at 380 nm to 430 nm with excitation at 310 nm [52]. 

### 3.6. Irradiations

Irradiations were carried out in a device equipped with six fluorescent tubes (Sylvania, F15 W/350BL) emitting polychromatic light between 300 and 500 nm with a maximum at 365 nm (Supplementary Figure S5). Ten mL of solutions were irradiated in a Pyrex glass reactor (14 mm i.d.) let open to air. Drugs (5 × 10^−6^ M) were irradiated alone in purified water buffered to pH 7, or in presence of WEOM (5 mgC L^−1^). Dark control experiments were also performed in order to determine whether adsorption of drugs on WEOM takes place. For this, starting solutions of each drug and WEOM (5 mgC L^−1^) covered with aluminum foil were kept in the dark and aliquots (5 mL) were withdrawn from the bottles after 1, 2 and 3 h to determine the drug concentration. Probe molecules (2,4,6-trimethylphenol at 5 × 10^−6^ M, furfuryl alcohol at 10^−4^ and 5 × 10^−5^ M, or terephthalic acid at 10^−5^ M) were also irradiated alone in pH 7 buffered purified water, or in presence of WEOM (5 mgC L^−1^). When drugs, or probe molecules were irradiated in the presence of WEOM, the reactants were mixed with vortex during 3 min, poured in the reactor and immediately after lamps were turned on. At given irradiation times, small aliquots were taken for HPLC analyses. These data were used to determine the initial rates of phototransformation. The number of photons received by the 10 mL of solutions in the cylindrical reactor was measured using a radiometer QE65000 from Ocean Optics coupled to chemical actinometry using metamitron [53]. The rate of light absorption of WEOM (5 mgC L^−1^), R_a_^WEOM^ was equal to (7.2 ± 0.7) × 10^−6^ E s^−1^ (Supplementary Figure S1) The screen effect of WEOM on drugs was calculated as described in SI-Text 1. All the rates of photodegradation obeyed an apparent first order kinetics, and the apparent first order reaction rate constants (k in s^−1^) were calculated according to Equation (4):

ln C_t_/C_0_ = −k × t (4)

where C_t_ is the concentration of the chemical at the irradiation time t and Co is the initial concentration. Irradiations were duplicated.

### 3.7. HPLC Analyses

HPLC analyses were carried out at 25 °C on an Alliance (Waters, Milford, MA, USA) apparatus equipped with a photodiode array detector (model 2998), fluorescence detector (model 2475) and two pumps (Waters 2695). Separation was achieved on a reverse phase Nucleodur, Macherey-Nagel C_8_ column (5 μm, 150 mm × 4.6 mm) equipped with a 4/3 pre-column made of the same material. The binary solvent system used was composed of solvent A (100% MeOH) and solvent B (water acidified by 0.03% of H_3_PO_4_). The best separation of psychoactive drugs was obtained with the following gradient: from 0–20 min, 20% A, then from 20–27 min, 80% A and return to 20% A. The solvent flow was 0.75 mL.min^−1^ and the volume injection was 25 μL. The eluent was a mixture of 20% MeOH and 80% water acidified with orthophosphoric acid (0.1%) for the experiments with terephthalic acid and furfuryl alcohol while a mobile phase of 50% acetonitrile and 50% acidified water was used for the experiments with 2,4,6-trimethylphenol. Hydroxyterephthalate concentration was measured by fluorescence (λ_exc_ = 320 nm and λ_em_ = 430 nm) using a calibration curve (Supplementary Figure S3). Analyses were duplicated.

Psychoactive drugs photoproducts were identified by HRMS performed on an Orbitrap Q-Exactive (Thermo Scientific, Waltham, MA, USA) coupled to an UHPLC Ultimate 3000 RSLC (Thermo Scientific, Waltham, MA, USA) equipped with an Acquity Phenomenex (2.1 mm × 100 mm, 1.7 μm particle size) analytical column (Waters, Milford, MA, USA). The aqueous solvent (A) consisted of a mixture of 0.1% formic acid and the organic phase (B) was acetonitrile. The separation was achieved with a gradient program consisting of 0–7.5 min 5%, 7.5–8.5 min 99% of the mobile phase B. After 8.5 min the gradient was returned to the initial conditions and analytical column was reconditioned for 3.5 min. The flow rate was set to 0.45 mL.min^−1^. The injection volume was 20 μL. The mass spectrometer operated in the positive and negative (ESI) electrospray ionization mode. The system was controlled by Xcalibur 2.2 (Thermo Fisher Scientific software, Waltham, MA, USA). The spray voltage was 3 kV for the positive and negative mode. For all proposed elemental formula, the error between the measured mass and the exact mass was less than 5 ppm.

### 3.8. Ecotoxicity Assessment

The ecotoxicity of the 3 target analytes and their by-products were predicted using ECOSAR program (v 2.0). ECOSAR uses a quantitative structure-activity relationship approach to predict the toxicity of a molecule based on its structure. The relevant endpoints are the acute toxicity, LC_50_ (concentration of tested compound that is lethal to half of fish and daphnia population after 96 h and 48 h of exposure, respectively) and EC_50_ (concentration of tested compound that inhibits the growth % of green algae after 96 h of exposure). The chronic toxicity values (ChV) of the drugs and their by-products also were predicted using the same program, for freshwater fish, daphnid and algae as well. Concerning accuracy, a compound is considered more toxic than another if the expected values vary by at least an order of magnitude [54]. The program (v 2.0) is freely available at the website: https://www.epa.gov/tsca-screening-tools/ecological-structure-activity-relationships-ecosar-predictive-model (accessed on 1 March 2021).

## 4. Conclusions

We showed that the water-soluble organic constituents of sediments are able to induce the oxidation of drugs under simulated solar light. The scavenging techniques revealed that hydroxyl radicals were the major contributors of these oxidations even though irradiation of WEOM led to other oxidant species. Thirty-five photoproducts were detected and identified by means of high-resolution mass spectrometry. Some of the proposed degradation pathways are found to be in common with all three drugs (oxidation through O addition or substitution of the halogen by OH), some are shared by only two of them (ring detachment or N-demethylation), while others are specific to a particular drug (dehydrogenation, N-elimination or ring opening). This study demonstrates that the fate of sertraline, clozapine and citalopram in lakes can be affected by sedimental organic constituents through photodegradation and that many by-products potentially toxic can be formed. Based on ECOSAR software, ecotoxicity assessments showed that toxic and very toxic by-products can be produced for sertraline, while harmful and not harmful TPs could be formed after WEOM mediated photodegradation of citalopram and clozapine.

## Figures and Tables

**Table 1:** HRMS data of the main photoproducts of SER after 8 h of irradiation in the presence of WEOM. Percentage of conversion = 69%.

Peak Code	Elemental Formula of [M + H^+^]^+^	*m*/*z*	TIC Peak Area	Chemical Modifications vs. SER
SER	C_17_H_18_Cl_2_N	306.0803 308.0771 310.0734		
SER-1 *Several* *peaks*	C_17_H_18_Cl_2_NO	322.0753 324.0722 326.0687	3.2 × 10^8^	SER + O fragment M − H_2_O
SER-1bis *Several* *peaks*	C_17_H_18_Cl_2_NO	322.0753 324.0722 326.0687		SER + O
SER-2 *Several* *peaks*	C_17_H_18_Cl_2_NO_2_	338.0699 340.0670 342.0641	1.7 × 10^7^	SER + 2O fragment M − H_2_O for some of the peaks
SER-3 *2 peaks*	C_17_H_16_Cl_2_N	304.0647 306.0615 308.0581	4.3 × 10^7^	SER − 2H
SER-4	C_16_H_13_Cl_2_O	291.0330 293.0300 295.0266	9.8 × 10^7^	SER − CH_3_NH_2_ + O
SER-5 *2 peaks*	C_17_H_19_ClNO	288.1146 290.1110	3.7 × 10^7^	SER − Cl + OH
SER-6	C_17_H_20_NO_2_	270.1481	1.8 × 10^6^	SER − 2Cl + OH + OH
SER-7	C_16_H_14_ClO	257.0721 259.0690	1.8 × 10^7^	SER − CH_3_NH_2_ + O − Cl + H
	Elemental formula of [M-H^+^]^−^	*m*/*z*	TIC peak Area	Chemical modifications vs. SER
SER-8	C_6_H_3_Cl_2_O	160.9553 162.9524 164.9495	5.7 × 10^6^	3,4-dichlorophenol
SER-9	C_7_H_3_Cl_2_O_2_	188.9507 190.9275 192.9279	2.6 × 10^6^	3,4-dichlorobenzoic acid

**Table 2:** HR-MS data of the main photoproducts of CLO after 8 h of irradiation in the presence of WEOM. Percentage of conversion = 44%.

Peak Code	Elemental Formula of [M + H^+^]^+^	*m*/*z*	TIC Peak Area	Chemical Modification vs. CLO
CLO	C_18_H_20_ClN_4_	327.1375 329.1341		
CLO-1 *Several* *peaks*	C_18_H_20_ClN_4_O	343.1323 345.1291	1.4 × 10^8^	ClO + O
CLO-2 *Several* *peaks*	C_18_H_20_ClN_4_O_2_	359.1273 361.1238	1.5 × 10^7^	CLO + 20
CLO-3 *Several* *peaks*	C_18_H_20_ClN_4_O_3_	375.1226 377.1193	2 × 10^6^	CLO + 3O
CLO-4	C_17_H_18_ClN_4_	313.1218 315.1186	5.6 × 10^7^	CLO − CH_2_
CLO-5	C_16_H_18_ClN_4_	301.1216	6.8 × 10^7^	CLO − C_2_H_2_
CLO-6	C_18_H_21_N_4_O	309.1716	1 × 10^6^	CLO − Cl + OH
CLO-7	C_17_H_18_ClN_4_O	329.1166 331.1136	1.2 × 10^7^	CLO − Cl + H − CH_2_
CLO-8	C_17_H_19_N_4_O_3_	327.1455	3.2 × 10^6^	CLO − CH_2_ − Cl + OH + 2O
CLO-9	C_18_H_19_N_4_O_2_	323.1504	4 × 10^6^	CLO − Cl + OH − 2H + O

**Table 3:** HR-MS data of the main photoproducts of CIT after 8 h of irradiation in the presence of WEOM. Percentage of conversion = 33%.

Peak Code	Elemental Formula of [M + H^+^]^+^	*m*/*z*	TIC peak Area	Chemical Modification vs. CIT
CIT	C_20_H_22_ON_2_F	325.1714		
CIT-1 *Several* *peaks*	C_20_H_22_O_2_N_2_F	341.1658	1.3 × 10^9^	CIT + O fragment M − H_2_O
CIT-1 bis *Several* *peaks*	C_20_H_22_O_2_N_2_F	341.1658		CIT + O
CIT-2	C_20_H_20_O_2_N_2_F	339.1506	7.2 × 10^8^	CIT − 2H + O
CIT-3	C_20_H_20_O_3_N_2_F	355.1456	1.3 × 10^7^	CIT − 2H + 2O
CIT-4	C_19_H_20_ON_2_F	311.1560	2 × 10^7^	CIT − CH_2_
CIT-5	C_20_H_23_O_2_N_2_	323.1757	2.5 × 10^8^	CIT − F + OH
CIT-6	C_14_H_19_ON_2_	231.1492	1 × 10^7^	CIT − aromatic ring
CIT-7 *Several* *peaks*	C_14_H_17_O_2_N_2_	245.1287	2.2 × 10^8^	CIT − aromatic ring-2H+O
CIT-8	C_19_H_20_O_2_N_2_F	327.1509	2 × 10^8^	CIT − CH_2_ + O
CIT-9	C_19_H_20_O_3_N_2_F	343.1456	1 × 10^7^	CIT − CH_2_ + 2O
CIT-10	C_19_H_18_O_2_N_2_F	325.1350	1.2 × 10^8^	CIT − CH_2_- − 2H + O
CIT-11	C_20_H_21_O_4_N_2_	353.1495	8.3 × 10^6^	CIT − F-2H + OH + 2O
CIT-12	C_20_H_21_O_3_N_2_	337.1546	1.1 × 10^7^	CIT − F+OH − 2H
CIT-13	C_20_H_23_O_3_N_2_	339.1703	1.2 × 10^7^	CIT − F+OH +O
CIT-14 *Several* *peaks*	C_19_H_21_O_4_N_2_	341.1491	1 × 10^7^	CIT − CH_2_ − F+OH+2O
CIT-15	C_19_H_19_O_3_N_2_	323.1393	1.3 × 10^6^	CIT − CH_2_ − F+OH − 2H+O

**Table 4:** Toxicity classification according to the Globally Harmonized System of Classification and Labelling of Chemicals (GHS). (United Nations, 2011).

Toxicity Range [mg/L]	Class
LC_50_/EC_50_/ChV ≤ 1	Very toxic
1 > LC_50_/EC_50_/ChV ≤ 10	Toxic
10 > LC_50_/EC_50_/ChV ≤ 100	Harmful
LC_50_/EC_50_/ChV > 100	Not harmful

**Table 5:** Toxicity predictions for SER and its transformation products using ECOSAR software.

	Acute Toxicity [mg/L]			Chronic Toxicity (ChV) [mg/L]		
	Fish (LC50)	Daphnid (LC50)	Algae (EC50)	Fish (LC50)	Daphnid (LC50)	Algae (EC50)
SER	0.408	0.071	0.028	0.0074	0.0085	0.012
SER-1	0.887	0.147	0.063	0.019	0.017	0.027
SER-2	1.93	0.301	0.154	0.049	0.032	0.059
SER-3	0.078	0.132	0.00072	0.0018	0.021	0.019
SER-4	0.477	0.357	0.839	0.065	0.075	0.408
SER-5	2.10	0.322	0.162	0.0057	0.034	0.064
SER-6	10.8	1.45	0.942	0.435	0.134	0.341
SER-7	1.59	1.13	2.07	0.201	0.201	0.879
SER-8	6.24	3.88	0.566	0.671	0.489	1.46
SER-9	5.96	3.92	5.31	0.692	0.572	1.92

**Table 6:** Toxicity predictions for CLO and its transformation products using ECOSAR software.

	Acute Toxicity [mg/L]			Chronic Toxicity (ChV) [mg/L]		
	Fish (LC50)	Daphnid (LC50)	Algae (EC50)	Fish (LC50)	Daphnid (LC50)	Algae (EC50)
CLO	17.7	2.32	1.58	0.764	0.210	0.563
CLO-1	65.2	20.1	6.40	4.58	2.20	8.65
CLO-2	141	15.9	14.6	9.71	1.24	4.67
CLO-3	518	53.1	59.1	47.9	3.76	17.6
CLO-4	23.3	2.98	2.13	1.09	0.263	0.746
CLO-5	26.0	3.29	2.41	1.25	0.287	0.835
CLO-6	155	17.2	16.4	11.3	1.31	5.17
CLO-7	86.0	10.0	8.66	5.38	0.804	2.83
CLO-8	1.65E+3	153	207	206	9.82	57.3
CLO-9	683	67.7	80.5	69.8	4.65	23.4

**Table 7:** Toxicity predictions for CIT and its transformation products using ECOSAR software.

	Acute Toxicity [mg/L]			Chronic Toxicity (ChV) [mg/L]		
	Fish (LC50)	Daphnid (LC50)	Algae (EC50)	Fish (LC50)	Daphnid (LC50)	Algae (EC50)
CIT	4.47	0.652	0.360	0.140	0.065	0.138
CIT-1	48.0	5.86	4.62	2.60	0.493	1.56
CIT-2	21.3	2.77	1.93	0.954	0.247	0.680
CIT-3	249	26.9	27.0	19.7	2.01	8.35
CIT-4	5.88	0.838	0.486	0.199	0.082	0.183
CIT-5	61.6	7.35	6.06	3.58	0.604	2.01
CIT-6	37.2	4.50	3.62	2.08	0.374	1.21
CIT-7	260	27.2	29.1	22.7	1.97	8.80
CIT-8	56.7	6.81	5.54	3.23	0.564	1.85
CIT-9	330	34.7	36.6	28.0	2.53	11.1
CIT-10	28.1	3.56	2.60	1.36	0.310	0.903
CIT-11	636	63.8	74.1	62.6	4.44	21.7
CIT-12	294	31.2	32.	24.4	2.29	9.88
CIT-13	134	15.1	13.9	9.22	1.17	4.43
CIT-14	918	89.3	110	99.2	6.02	31.6
CIT-15	176	19.4	18.8	13.1	1.48	5.88

## Supplementary Materials

The following are available online, Figure S1: Photodegradation of 2,4,6-trimethylphenol in the presence of WEOM, Figure S2: Photodegradation of furfuryl alcohol in the presence of WEOM, Figure S3: Formation of hydroxyterephthalic acid upon irradiation of terephthalic acid in the presence of WEOM, Figure S4: Adsorption of drugs to sediment, Figure S5: Irradiance of the fluorescent tubes, Table S1: Rate constants of drugs photodegradation in the presence of WEOM; Text Section S1: Screen effect calculation.

## Sample Availability

Samples of sediments are available from the authors.
