# Supplementary material for: Phototransformation of Three Psychoactive Drugs in Presence of Sedimental Water Extractable Organic Matter
Source: Molecules. 2021 Apr 23;26(9):2466. doi: 10.3390/molecules26092466 (PMC8122907; doi:10.3390/molecules26092466)

Supporting Information

# Fate of three psychoactive drugs in Lake Pamvotis: adsorption on sediment and photodegradation in presence of sedimental water extractable organic matter

Cristiana Jiménez-Holgádo[1,2], Vasilios Sakkas[1], Claire Richard[2*]

[1] Laboratory of Analytical Chemistry, Department of Chemistry, School of Sciences, University of Ioannina,45110 Ioannina, Greece

[2] Université Clermont Auvergne, CNRS, SIGMA-Clermont, ICCF, F-63000 Clermont-Ferrand, France

Table S1: Rate constants of photodegradation of SER, CLO and CIT in water ($k_{direct\ photolysis}$), in the presence of WEOM (k), and in the presence of WEOM+2-propanol ($k_{2\text{-}propanol}$). $k_{direct\ photolysis*}$ corresponds to $k_{direct\ photolysis}$ after correction for the screen effect of WEOM

| $k(s^{-1})$ | Sertraline | Clozapine | Citalopram |
|---|---|---|---|
| $k_{direct\ photolysis}$ | $1.6\times10^{-5}$ | $0.60\times10^{-5}$ | $0.13\times10^{-5}$ |
| $k_{direct\ photolysis*}$ | $1.4\times10^{-5}$ | $0.53\times10^{-5}$ | $0.11\times10^{-5}$ |
| $k= k_{direct\ photolysis*}+ k_{WEOM}$ | $3.2\times10^{-5}$ | $2.5\times10^{-5}$ | $2.1\times10^{-5}$ |
| $k_{WEOM}$ | $1.8\times10^{-5}$ | $2.0\times10^{-5}$ | $2.0\times10^{-5}$ |
| $k_{2\text{-}propanol}$ | $0.49\times10^{-5}$ | $0.46\times10^{-5}$ | $0.44\times10^{-5}$ |

Section S1 : Screen effect calculation

The rate of light absorption by drugs ($R_a^d$) are calculated using the following Eq. SI-1 when they are alone:

$$R_a^d = \sum_{\lambda=290}^{\lambda=500} I_0^\lambda \ (1 - 10^{-A^\lambda d}) \qquad \text{Eq. SI-1}$$

where $I_0^\lambda$ is the amount of photons received by the samples for the wavelength range λ - λ+ 5 nm and $A^\lambda d$ is the absorbance of the drug solution averaged for the same wavelength range. The rate of light absorption by WEOM ($R_a^{WEOM}$) is also calculated using Eq. SI-1 by replacing $A^\lambda d$ by $A^\lambda WEOM$.

In the presence of WEOM, the rate of light absorption by the drugs, $R_{a\,WEOM}^d$ becomes:

$$R_{a\,WEOM}^d = \sum_{\lambda=290}^{\lambda=500} \frac{A^\lambda d}{A^\lambda d + A^\lambda WEOM} I_0^\lambda \ (1 - 10^{-(A^\lambda d + A^\lambda WEOM)}) \qquad \text{Eq. SI-2}$$

The apparent first order reaction rate constants $k_{direct\ photolysis*}$ of drug disappearance in presence of WEOM are therefore equal to:

$$k_{direct\ photolysis*} = k_{direct\ photolysis} \times R_{a\,WEOM}^d / I_a^d \qquad \text{Eq. SI-3}$$

Figure S1: Photodegradation of 2,4,6-trimethylphenol ($5\times10^{-6}$ M) upon irradiation in the presence of WEOM (5 mgC.$L^{-1}$) at pH=7. The slope corresponding to the apparent first order reaction rate constant is equal to k= 0.020±0.002 $min^{-1}$.

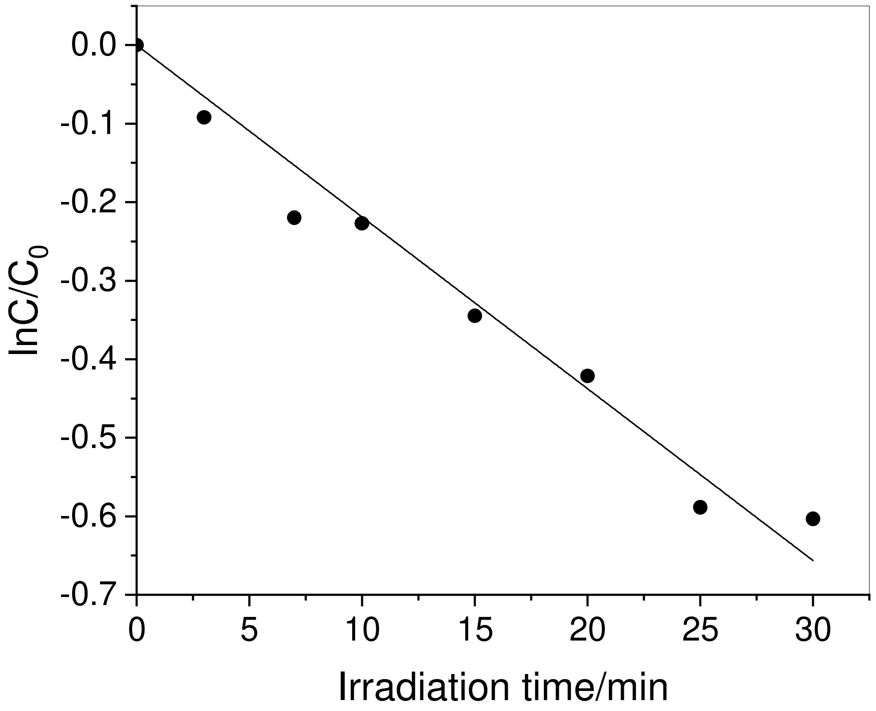

Figure S2: Photodegradation of furfuryl alcohol ($10^{-4}$ M and $5\times10^{-5}$ M) upon irradiation in the presence of WEOM (5 mgC.$L^{-1}$) at pH=7. The slope corresponding to the apparent first order reaction rate constant is equal to k= 0.0011±0.002 $min^{-1}$.

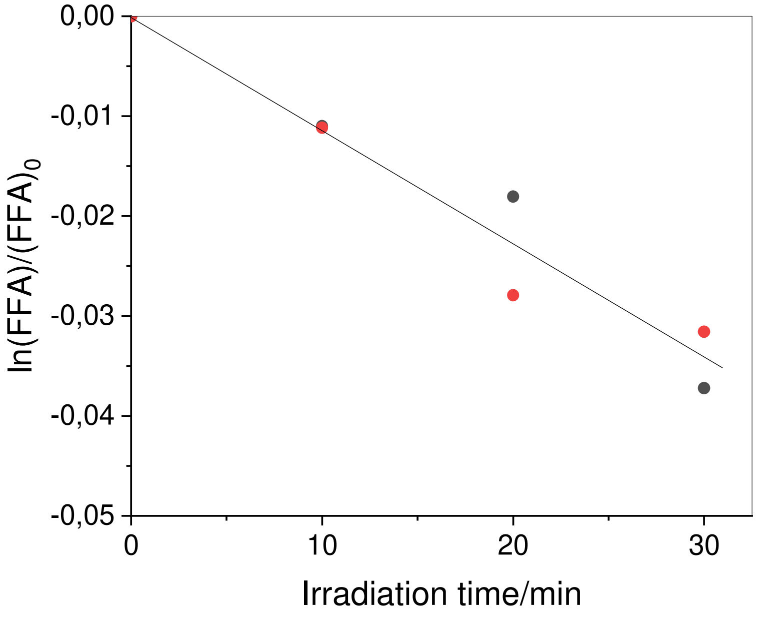

Figure S3: Calibration curve of hydroxyterephthalic acid (TAOH) obtained by UHPLC-HRPS analyses (A) and formation profile of TAOH upon irradiation of terephthalate ($10^{-5}$ M) in the presence of WEOM (5 mgC.$L^{-1}$) at pH=7 (B). TAOH is formed by reaction of terephthalate with HO· radicals.

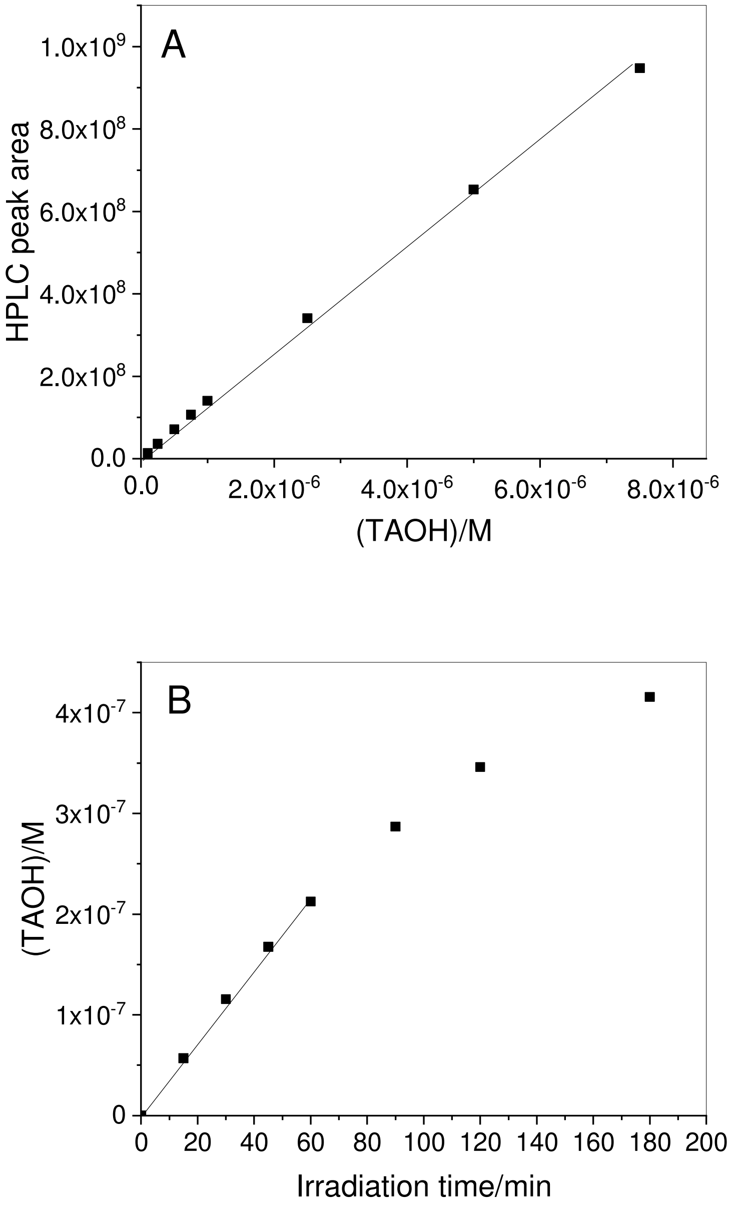

Figure S4: Adsorption of SER (▲), CLO (●) and CIT (○) (0.27-0.29 mg) to sediment. Percentage of adsorbed drug against the amount of sediment in suspension (A) and logarithmic representation of Freundlich equation (B)

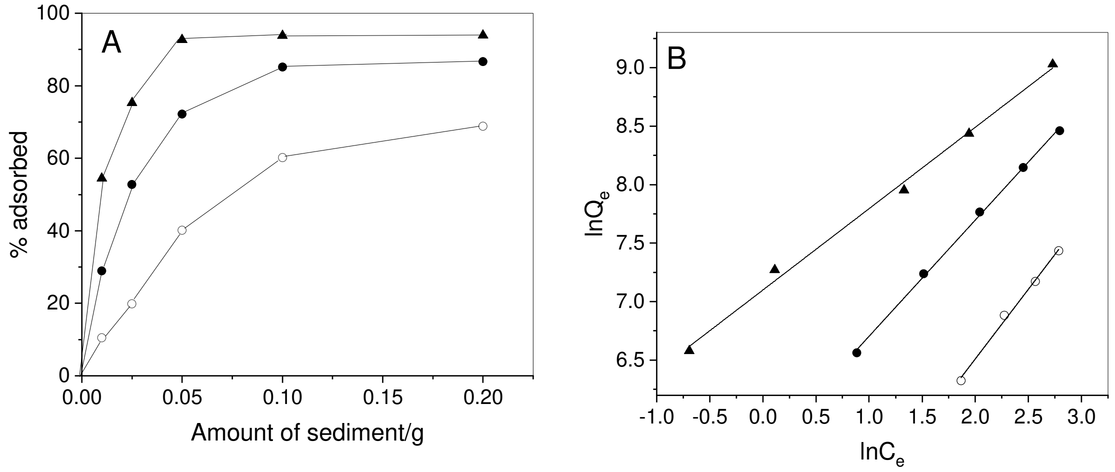

Figure S5: Irradiance of the fluorescent tubes

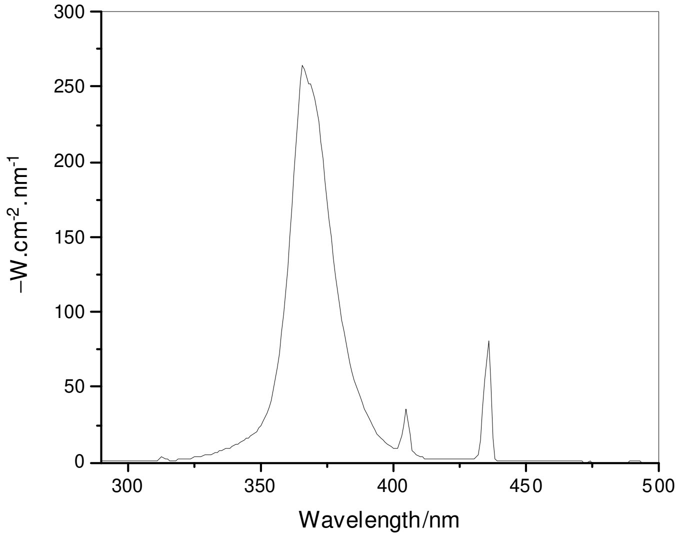

Supplement: Supplementary file 1 [file molecules-26-02466-s001.zip › molecules-1177878-supplementary.pdf]
